# Supplementary material for: Allosteric regulation and crystallographic fragment screening of SARS-CoV-2 NSP15 endoribonuclease
Source: Nucleic Acids Res. 2023 Apr 28;51(10):5255–70. doi: 10.1093/nar/gkad314 (PMC10250223; doi:10.1093/nar/gkad314)
Supplement: gkad314_Supplemental_Files [file gkad314_supplemental_files.zip › Supplementary datasets.zip!/159/Refine_4/01-BUSTER/Cycle-4/shell.01/R_Rfree.html]

# Rxpct factors in resolution bins at cycle 0


|  |  |  |  |  |  |  |  |  |
| --- | --- | --- | --- | --- | --- | --- | --- | --- |
|  | **<d> (Å)**  Rxpct\_Work | Rxpct\_Free | Rxpct\_All | |  |  |  |  |
|  | 12.8357 | 0.3297 | 0.4262 | 0.3337 |  |
|  | 7.4107 | 0.2748 | 0.2966 | 0.2761 |  |
|  | 5.7403 | 0.2698 | 0.3008 | 0.2710 |  |
|  | 4.8514 | 0.2075 | 0.2348 | 0.2090 |  |
|  | 4.2786 | 0.2016 | 0.1980 | 0.2014 |  |
|  | 3.8701 | 0.2112 | 0.2388 | 0.2126 |  |
|  | 3.5600 | 0.2263 | 0.2535 | 0.2276 |  |
|  | 3.3142 | 0.2299 | 0.2729 | 0.2325 |  |
|  | 3.1131 | 0.2249 | 0.2392 | 0.2256 |  |
|  | 2.9447 | 0.2125 | 0.2172 | 0.2127 |  |
|  | 2.8010 | 0.2053 | 0.2097 | 0.2055 |  |
|  | 2.6764 | 0.1978 | 0.2092 | 0.1984 |  |
|  | 2.5671 | 0.1907 | 0.1969 | 0.1910 |  |
|  | 2.4702 | 0.1839 | 0.1887 | 0.1841 |  |
|  | 2.3835 | 0.1819 | 0.1977 | 0.1826 |  |
|  | 2.3054 | 0.1719 | 0.1930 | 0.1729 |  |
|  | 2.2344 | 0.1850 | 0.1976 | 0.1856 |  |
|  | 2.1696 | 0.1887 | 0.1972 | 0.1892 |  |
|  | 2.1102 | 0.1990 | 0.1979 | 0.1989 |  |
|  | 2.0554 | 0.2228 | 0.2124 | 0.2223 |  |
|  | **Overall** | 0.2202 | 0.2399 | 0.2212 |  |

# Rxpct factors in resolution bins at cycle 1


|  |  |  |  |  |  |  |  |  |
| --- | --- | --- | --- | --- | --- | --- | --- | --- |
|  | **<d> (Å)**  Rxpct\_Work | Rxpct\_Free | Rxpct\_All | |  |  |  |  |
|  | 12.8357 | 0.3300 | 0.4267 | 0.3340 |  |
|  | 7.4107 | 0.2749 | 0.2968 | 0.2762 |  |
|  | 5.7403 | 0.2695 | 0.3006 | 0.2707 |  |
|  | 4.8514 | 0.2073 | 0.2350 | 0.2088 |  |
|  | 4.2786 | 0.2012 | 0.1978 | 0.2010 |  |
|  | 3.8701 | 0.2107 | 0.2385 | 0.2121 |  |
|  | 3.5600 | 0.2255 | 0.2530 | 0.2269 |  |
|  | 3.3142 | 0.2289 | 0.2723 | 0.2315 |  |
|  | 3.1131 | 0.2239 | 0.2386 | 0.2246 |  |
|  | 2.9447 | 0.2117 | 0.2165 | 0.2120 |  |
|  | 2.8010 | 0.2046 | 0.2098 | 0.2049 |  |
|  | 2.6764 | 0.1972 | 0.2093 | 0.1978 |  |
|  | 2.5671 | 0.1904 | 0.1968 | 0.1908 |  |
|  | 2.4702 | 0.1839 | 0.1893 | 0.1842 |  |
|  | 2.3835 | 0.1819 | 0.1977 | 0.1826 |  |
|  | 2.3054 | 0.1721 | 0.1938 | 0.1731 |  |
|  | 2.2344 | 0.1852 | 0.1976 | 0.1858 |  |
|  | 2.1696 | 0.1889 | 0.1974 | 0.1894 |  |
|  | 2.1102 | 0.1991 | 0.1984 | 0.1991 |  |
|  | 2.0554 | 0.2230 | 0.2131 | 0.2224 |  |
|  | **Overall** | 0.2198 | 0.2398 | 0.2208 |  |

# Rxpct factors in resolution bins at cycle 2


|  |  |  |  |  |  |  |  |  |
| --- | --- | --- | --- | --- | --- | --- | --- | --- |
|  | **<d> (Å)**  Rxpct\_Work | Rxpct\_Free | Rxpct\_All | |  |  |  |  |
|  | 12.8357 | 0.3303 | 0.4272 | 0.3343 |  |
|  | 7.4107 | 0.2749 | 0.2970 | 0.2763 |  |
|  | 5.7403 | 0.2691 | 0.3003 | 0.2703 |  |
|  | 4.8514 | 0.2069 | 0.2351 | 0.2084 |  |
|  | 4.2786 | 0.2007 | 0.1975 | 0.2006 |  |
|  | 3.8701 | 0.2101 | 0.2382 | 0.2115 |  |
|  | 3.5600 | 0.2247 | 0.2525 | 0.2260 |  |
|  | 3.3142 | 0.2280 | 0.2717 | 0.2306 |  |
|  | 3.1131 | 0.2229 | 0.2382 | 0.2236 |  |
|  | 2.9447 | 0.2110 | 0.2159 | 0.2112 |  |
|  | 2.8010 | 0.2039 | 0.2098 | 0.2042 |  |
|  | 2.6764 | 0.1967 | 0.2096 | 0.1974 |  |
|  | 2.5671 | 0.1902 | 0.1968 | 0.1906 |  |
|  | 2.4702 | 0.1840 | 0.1899 | 0.1843 |  |
|  | 2.3835 | 0.1820 | 0.1978 | 0.1827 |  |
|  | 2.3054 | 0.1724 | 0.1942 | 0.1734 |  |
|  | 2.2344 | 0.1854 | 0.1976 | 0.1859 |  |
|  | 2.1696 | 0.1892 | 0.1977 | 0.1896 |  |
|  | 2.1102 | 0.1994 | 0.1987 | 0.1993 |  |
|  | 2.0554 | 0.2231 | 0.2134 | 0.2226 |  |
|  | **Overall** | 0.2194 | 0.2397 | 0.2204 |  |

# Rxpct factors in resolution bins at cycle 3


|  |  |  |  |  |  |  |  |  |
| --- | --- | --- | --- | --- | --- | --- | --- | --- |
|  | **<d> (Å)**  Rxpct\_Work | Rxpct\_Free | Rxpct\_All | |  |  |  |  |
|  | 12.8357 | 0.3311 | 0.4350 | 0.3355 |  |
|  | 7.4107 | 0.2692 | 0.3038 | 0.2713 |  |
|  | 5.7403 | 0.2505 | 0.2834 | 0.2517 |  |
|  | 4.8514 | 0.1923 | 0.2215 | 0.1939 |  |
|  | 4.2786 | 0.1872 | 0.1901 | 0.1874 |  |
|  | 3.8701 | 0.1951 | 0.2337 | 0.1970 |  |
|  | 3.5600 | 0.2087 | 0.2411 | 0.2102 |  |
|  | 3.3142 | 0.2100 | 0.2540 | 0.2127 |  |
|  | 3.1131 | 0.2074 | 0.2342 | 0.2087 |  |
|  | 2.9447 | 0.1996 | 0.2159 | 0.2003 |  |
|  | 2.8010 | 0.1928 | 0.2105 | 0.1937 |  |
|  | 2.6764 | 0.1889 | 0.2079 | 0.1899 |  |
|  | 2.5671 | 0.1872 | 0.1932 | 0.1876 |  |
|  | 2.4702 | 0.1845 | 0.1914 | 0.1849 |  |
|  | 2.3835 | 0.1845 | 0.2080 | 0.1855 |  |
|  | 2.3054 | 0.1756 | 0.1999 | 0.1767 |  |
|  | 2.2344 | 0.1899 | 0.2075 | 0.1907 |  |
|  | 2.1696 | 0.1927 | 0.2028 | 0.1932 |  |
|  | 2.1102 | 0.2045 | 0.2045 | 0.2045 |  |
|  | 2.0554 | 0.2314 | 0.2203 | 0.2309 |  |
|  | **Overall** | 0.2094 | 0.2352 | 0.2107 |  |
